# The Fronto-Temporal Cortex Has Increased Subcortical Connectivity In Utero and Plasticity in Adulthood

**DOI:** 10.1523/JNEUROSCI.0832-25.2025

**Published:** 2026-01-21

**Authors:** Gabriela Epihova, Dimitar Z. Epihov, Danyal Akarca, Duncan E. Astle

**Affiliations:** ^1^MRC Cognition and Brain Sciences Unit, University of Cambridge, Cambridge, United Kingdom; ^2^Plants, Photosynthesis and Soil Cluster, School of Biosciences, University of Sheffield, United Kingdom; ^3^Electrical & Electronic Engineering, Imperial College London, London, United Kingdom; ^4^Department of Psychiatry, University of Cambridge, Cambridge, United Kingdom

**Keywords:** fronto-temporal cortex, glioma, plasticity, prenatal connectivity, transcriptomics

## Abstract

The adult cerebral cortex is a heterogeneous structure with prominent functional differences between regions. However, less is known about how different regions acquire and maintain their functionality. Here, we leveraged connectomes and brain transcriptomes from human fetal and adult brains of both sexes to investigate early and late differences between cortical regions. We show that at 24 postgestational weeks fronto-temporal regions are disproportionally connected to subcortical regions, highlighting their role in early integrative cortical-subcortical communication. In adulthood, fronto-temporal cortex has lower myelin content and exhibits lower expression of marker genes of perineuronal nets, while showing higher expression of undifferentiated progenitor cells markers. These results suggest that in the adult brain the function of fronto-temporal regions reflects a heightened state of plasticity, possibly to maximize flexible neural responses. In contrast, the function of parietal and occipital regions aligns with decreased plasticity needed to support stable neural dynamics. Linking physiology to pathology, we show that the greater plasticity of the fronto-temporal cortex is coupled to higher oncogenic vulnerability—frontal and temporal regions have greater incidence of gliomas and express higher levels of genes upregulated in glioma even in the absence of malignancy, suggesting a greater glioma-like normative expression state. Together, these findings highlight the divergent patterns of connectivity in utero and plasticity in adulthood between cortical regions and provide a framework in which functional differences across cortical regions reflect differences in connectivity and plasticity.

## Significance Statement

Here we leveraged fetal neuroimaging and adult brain transcriptomes to investigate early and late differences between cortical regions. We present new evidence that already at mid-prenatal development, the fronto-temporal lobes are disproportionally connected to subcortical regions, potentially reflecting an early route to their establishment as integrative cortical centers. In adulthood, the fronto-temporal cortex had increased plasticity of its connections and cellular state which was coupled to greater oncogenic vulnerability. The combination of increased early connectivity and long-term plasticity might serve to maximize flexible neural representations and support the domain-general function of fronto-temporal regions.

## Introduction

The adult cerebral cortex exhibits marked functional heterogeneity. High-level cortical regions support complex, integrative computations enabling flexible cognition, and context-dependent behavior, whereas low-level sensory regions support stability by faithfully mapping to the source of their activation (Mesulam, 1998; Margulies et al., 2016). However, we know less about how these regions acquire and maintain their distinctive functionality. A simple principle is that the functional properties of different cortical regions will be, to some extent, a consequence of the pattern of their structural connections laid early in development. However, little is known about the developmental mechanisms guiding the early divergence of cortical regions, and in particular, whether particular regions are connectivity hubs already during early brain development. In network neuroscience a connectivity hub is a brain region that exhibits a disproportionately large number of connections and thus plays a central integrative role in the organization of the brain network. Understanding the early signatures of divergent in utero development across cortical regions has widespread implications for their functional properties in the adult brain.

In addition to early developmental differences, the remarkable integrative properties of the higher order regions likely reflect differences in postnatal plasticity mechanisms that enable these regions to flexibly integrate information. Throughout postnatal development, there are two main processes that contribute to the reduction of neural plasticity: myelination (McGee et al., 2005; Xin et al., 2024) and the formation of perineuronal nets (PNNs; Fawcett et al., 2019; Reichelt et al., 2019). The well-established consequence of myelination is that it increases the speed of neural signals, thus supporting fast and stable communication. However, it also decreases neural plasticity and contributes to the closure of critical periods (McGee et al., 2005; Xin et al., 2024). This is caused by the dual role of fast communication: fast conduction times support stable signals, but the lack of delay in signal transduction inherently impedes flexibility as it prevents a region to recognize and integrate signal patterns across longer periods of time. Interestingly, association regions are the last one to myelinate (Baum et al., 2022; Sydnor et al., 2023) and even when fully myelinated have less myelin than sensorimotor regions (Glasser and van Essen, 2011).

The second main plasticity-repressing mechanism is PNNs. PNNs are extracellular matrix structures that preferentially surround parvalbumin-positive GABAergic inhibitory neurons and are responsible for synaptic stabilization by acting as electrostatic insulators (Fawcett et al., 2019; Reichelt et al., 2019). Similarly to myelin, PNNs limit plasticity by decreasing the ability of a neuron to store electrical charge across its membrane, thus forcing it to fire rapidly. In contrast to myelination, much less is known about differences between cortical regions in the abundance of PNNs in the human brain, likely due to the lack of imaging correlates via standard neuroimaging techniques. In the rat cortex association regions have fewer PNNs compared with sensorimotor regions (Galtrey and Fawcett, 2007). It is not known whether this pattern is evident in the human brain and when during development this difference emerges.

Here we asked: how do high-level cortical regions achieve its remarkable ability to integrate information, allowing it to support increasingly abstract levels of representation? We reasoned that an integrative region must have the following properties: (1) have more connections early in development to provide greater source of inputs, (2) keep these connections plastic for longer to maximize integrative communication and, (3) harbor an intrinsically more plastic cellular state. To that end, we first characterized fetal structural connectivity and gene expression to quantify regional differences in number of early connections and cell type markers. With respect to the second and third properties, we combined cortical myelin maps derived from T1w/T2w with brain transcriptomics to investigate whether in adulthood fronto-temporal regions maintain heightened state of plasticity. Finally, we linked physiology to pathology by exploring whether regions with heightened plasticity in adulthood harbor a higher oncogenic potential.

## Materials and Methods

### Diffusion-weighted imaging (DWI), fiber tracking, and connectome construction

Human fetal diffusion data was taken from the Developing Human Connectome Project (dHCP). The dHCP is a collaborative effort between King's College London, Imperial College London, and Oxford University that collects fetal and neonatal neuroimaging data. Detailed description of acquisition parameters and preprocessing steps is provided in Price et al. (2019) and Wilson et al. (2023). We restricted our analyses on fetal brains aged 23–25 postgestational weeks (*n* = 22) to avoid the effect of myelination on connectivity estimates in older subjects. The earliest that myelination has been microscopically observed is at 25 p.g. weeks with the first myelin sheaths appearing in the globus pallidus, pallido-thalamic fibers of the posterior internal capsule, and ventral lateral nucleus of the thalamus (Hasegawa et al., 1992). In utero imaging of fetuses of that age presents challenges, such as reduced anisotropy; however, unmyelinated white matter tracts still show signal intensity changes consistent with anisotropic water diffusion (Hüppi et al., 1998). Using dMRI previous work has successfully reconstructed major white matter tracts as early as 22 postgestational weeks (Wilson et al., 2021; Calixto et al., 2025). We validated anatomical tracts that are expected to be present at this developmental stage (Supp Fig. 1*a*,*b*) and compared their fractional anisotropy (FA) values to published values for similarly aged fetal samples. Our FA values were comparable with published FA values in that developmental period (Supp Fig. 1*c*).

Preprocessed scans were reconstructed in DSI studio (Yeh et al., 2010) via generalized q-sampling imaging using a sampling length ratio of 1.25 in native space. Deterministic fiber tracking was performed in DSI Studio with 1,000,000 seeds. Visualization of whole-brain fiber tractography across all subjects is shown in Supp Figure 1*d*. To prevent radial glia and spurious short fibers being incorrectly included in the connectivity estimates, we restricted the minimum fiber length to 30 mm. Streamlines resulting from the fiber tracking were parcellated with a fetal volumetric atlas that corresponded to the gestational age of the subject (either 23, 24, or 25 postgestational weeks) taken from http://crl.med.harvard.edu/research/fetal_brain_atlas/ (Gholipour et al., 2017). This atlas contains 78 distinct cortical parcellations. As short-range local connectivity between regions within the same lobe may not be reliable at the fetal stage of brain development, we modified the atlas by grouping all regions within their respective lobes into a single mask, which resulted in four cortical regions: frontal, temporal, parietal, and occipital. The subcortical regions included in the fetal atlas were the left and right hippocampus, parahippocampal region, amygdala, caudate, putamen, and thalamus.

After manual inspection, eight fetal scans were removed from the analyses for incorrect reconstruction and fitting of the atlas parcels to the anatomical structures/brain orientation. The remaining 14 fetuses had a mean age of 23.92 pgwk (range, 23.43–24.71), 8 male, 6 female.

The structural connectome of each subject was constructed by parcellating the whole-brain tractography with 16 regions (4 cortical + 12 subcortical regions) derived from the fetal atlas. The connectivity matrix was calculated by using the fiber density which represents the number of streamlines connecting each pair of regions. All connections within the same regions were excluded.

### Human fetal transcriptomics data and cell type enrichment analysis

RNAseq data was obtained from the publicly available BrainSpan Developing Brain atlas (https://www.brainspan.org), covering the period from 12 postgestational weeks to adulthood. The data used in the current analyses included gene expression from 11 cortical: dorsal frontal cortex (DFC), medial frontal cortex (MFC), orbito-frontal cortex (OFC), ventral frontal cortex (VFC), motor cortex (M1C), somato-sensory cortex (S1C), auditory cortex (A1C), inferior parietal cortex (IPC), superior temporal cortex (STC), inferior temporal cortex (ITC), and visual cortex (V1C). Data from corresponding regions in the left and right hemispheres were pooled together. The obtained gene expression data were in reads per kilobase per million (RPKM) values. To allow normalized comparisons across regions and timepoints, RPKM values were converted to transcripts per million (TPM) according to the formula:

$$\mathrm{TPM}=10^{6\;}*\;\frac{\mathrm{RPKM}}{\mathrm{sum}\;(\mathrm{RPKM})}.$$

A Uniform Manifold Approximation and Projection (UMAP) across the expression of all genes was performed for dimensionality reduction. The UMAP results indicated that at 37 weeks the expression patterns transition to a distinct state compared with earlier fetal and ex utero expression, replicating the previously reported transcriptomic transition beginning during late fetal development (10). As a result, and to facilitate for compatibility between our connectivity and transcriptomics analyses, we excluded data from 37 pgwk and constrained our analyses on the remaining early-to-mid fetal samples from 12 to 24 postgestational weeks (pgwk). This resulted in 13 donor samples (6 female, 7 male): 3 donors at 12 pgwk, 3 donors at 13 pgwk, 3 donors at 16 pgwk, 1 donor at 17 pgwk, 1 donor at 19 pgwk, 1 donor at 21 pgwk, 1 donor at 24 pgwk. As there was only one region to represent, the expression in the occipital lobe (V1) donors without expression data from V1 cortex was removed from the analysis to ensure no individual differences bias in expression.

First, we cross-referenced known brain cell type marker genes with a developmentally relevant single-cell RNAseq data which were derived from frontal cortex tissue across developmental timepoints from early fetal to adulthood (Zhu et al., 2023). Expression of maker genes were referenced across a set of predefined neuronal cell types: early fetal excitatory neurons (EN fetal early), late fetal excitatory neurons (EN fetal late), postnatal excitatory neurons (EN), fetal inhibitory neurons (IN fetal), medial ganglionic eminence-derived inhibitory neurons (IN-MGE), caudal ganglionic eminence-derived inhibitory neurons (IN-CGE), oligodendrocyte progenitor cells (OPC), oligodendrocytes, astrocytes, microglia, radial glia, intermittent progenitor cells (IPC), endothelial cells, pericytes, and vascular smooth muscle cells (VSMC). After ensuring that the marker genes are expressed uniquely in a cell type, we investigated whether expression of these markers in the bulk RNA samples from the BrainSpan Developing Brain atlas differed between the cortical lobes.

### Cortical myelin map, plasticity-related, and glioma-upregulated gene expression in adulthood

T1w/T2w cortical myelin maps were taken from the Human Connectome Project (Glasser et al., 2016) and parcellated with the Desikan–Killiany (DK) cortical atlas (Desikan et al., 2006) to derive a cortical myelination value for each region of the atlas. Normalized gene expression maps were taken from The Allen Human Brain Atlas (AHBA) using the *abagen* toolbox (Markello et al., 2021) and were also parcellated with the DK cortical atlas.

When comparing the four cortical lobes, the following DK regions were included in each lobe: (1) frontal: caudal anterior cingulate, caudal middle frontal, frontal pole, lateral orbito-frontal, medial orbito-frontal, pars opercularis, pars orbitalis, pars triangularis, rostral anterior cingulate, rostral middle frontal, superior frontal; (2) temporal: bankssts, entorhinal, fusiform, inferior temporal, middle temporal, parahippocampal, superior temporal, temporal pole, transverse temporal; (3) parietal: inferior parietal, isthmus cingulate, paracentral, posterior cingulate, precuneus, superior parietal, supramarginal; and (4) occipital: cuneus, lateral occipital, lingual, pericalcarine, inferior parietal, isthmus cingulate, paracentral, posterior cingulate, precuneus, superior parietal, supramarginal. Regions from the atlas not included in the cortical lobe classification were the insula, precentral, and postcentral.

There were 451 upregulated genes in glioblastoma identified in Neftel et al. (2019). In total, 325 of these genes matched to genes in the AHBA transcriptome, and 221 matched to genes in the BrainSpan atlas after removing the genes with low average expression values (<1 TPM). For each of the matching genes, we *z*-scored the expression levels across the cortical regions and then averaged the *z* scores of regions across the four cortical lobes.

## Results

### Structural connectivity differences across the cortex in the mid-prenatal period

**Figure 1.**
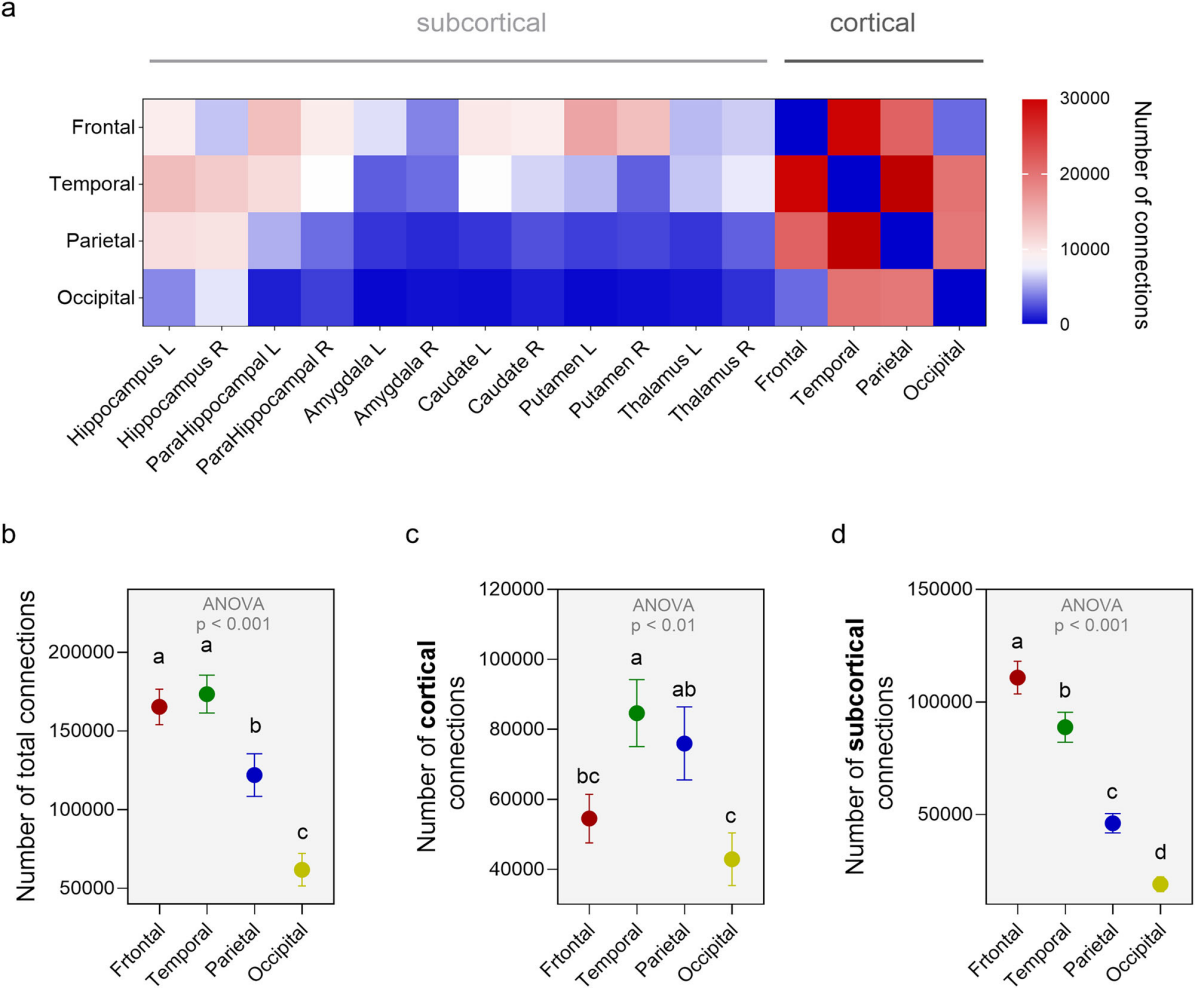
Structural brain connectivity at 24 postgestational weeks. ***a***, Average number of connections between each cortical lobe with the other lobes and subcortical regions. ***b***, Number of connections between each cortical lobe and all regions in the atlas. ***c***, Number of connections between each cortical lobe and the other three cortical lobes. ***d***, Number of connections between each cortical lobe and all subcortical regions in the atlas. Error bars represent ±1 SEM.

A simple first step toward understanding very early differences in connectivity between cortical regions is to look at differences in connection numbers early in development. We measured in utero brain connectivity from fetal diffusion MRI scans (dMRI) in 14 healthy fetuses from the Developing Human Connectome Project (dHCP). To avoid the potential effect of myelination beginning after 25 postgestational weeks (p.g. wks), we focused on the youngest available fetal brains scan aged 23–25 p.g. wks: mean age of 23.92 p.g. wks. For each individual we performed whole-brain fiber tracking and constructed individual connectomes by parcellating the whole-brain tractography with a fetal atlas of the corresponding age. The pattern of connectivity between regions was consistent across individuals as indicated by an average between-participants correlation of connectomes of *r* = 0.77 (Supp Fig. 2*a*,*b*). We averaged the individual connectivity matrices and compared the connections between each cortical lobe with the rest of the lobes and subcortical regions in the atlas (Fig. 1*a*). Next, to statistically compare the number of connections between the four lobes, for each individual we calculated the total number of connections, the cortical (between-lobe) connections, and the subcortical connections of each cortical lobe.

The frontal and temporal lobes had significantly more total connections than the parietal and occipital (ANOVA with FDR-corrected multiple comparisons: *F*_(3,52)_ = 18.61, *p* < 0.001; Fig. 1*b*). This pattern was primarily driven by the higher number of subcortical connections to the frontal and temporal lobes (ANOVA with FDR-corrected multiple comparisons: *F*_(3,52)_ = 54.30, *p* < 0.001; Fig. 1*d*). The increased connectivity of the frontal and temporal lobes to the subcortex was evident across all subcortical regions in the atlas and cannot be attributed to a single strong subcortical connection (Fig. 1*a*) or the physical distance between the lobes and subcortical structures (Supp Fig. 3*e*,*f*). The increased fronto-temporal connectivity to subcortical regions was consistent in all of the 14 individual subjects (Supp Fig. 2*c*) and present when the number of subcortical connections to each lobe was normalized by the voxel size of each cortical mask (Supp Fig. 3). However, although both the frontal and temporal lobes were enriched in subcortical connections, there were some differences in their pattern of subcortical connectivity. The increased fronto-subcortical connectivity was driven by a higher number of connections with the basal ganglia (caudate and putamen), whereas the increased temporo-subcortical connectivity was driven by connection between the temporal cortex and hippocampus (Fig. 1*a*).

There was also a significant difference between the lobes in the number of connections to cortical regions, with the highest cortical connectivity in the temporal and parietal lobes ANOVA with FDR-corrected multiple comparisons: *F*_(3,52)_ = 4.83, *p* = 0.005 (Fig. 1*b*), but this effect was not as strong and consistent across all subjects (Supp Fig. 2*c*).

### Frontal and temporal cortices were enriched in inhibitory neurons in the mid-prenatal period

**Figure 2.**
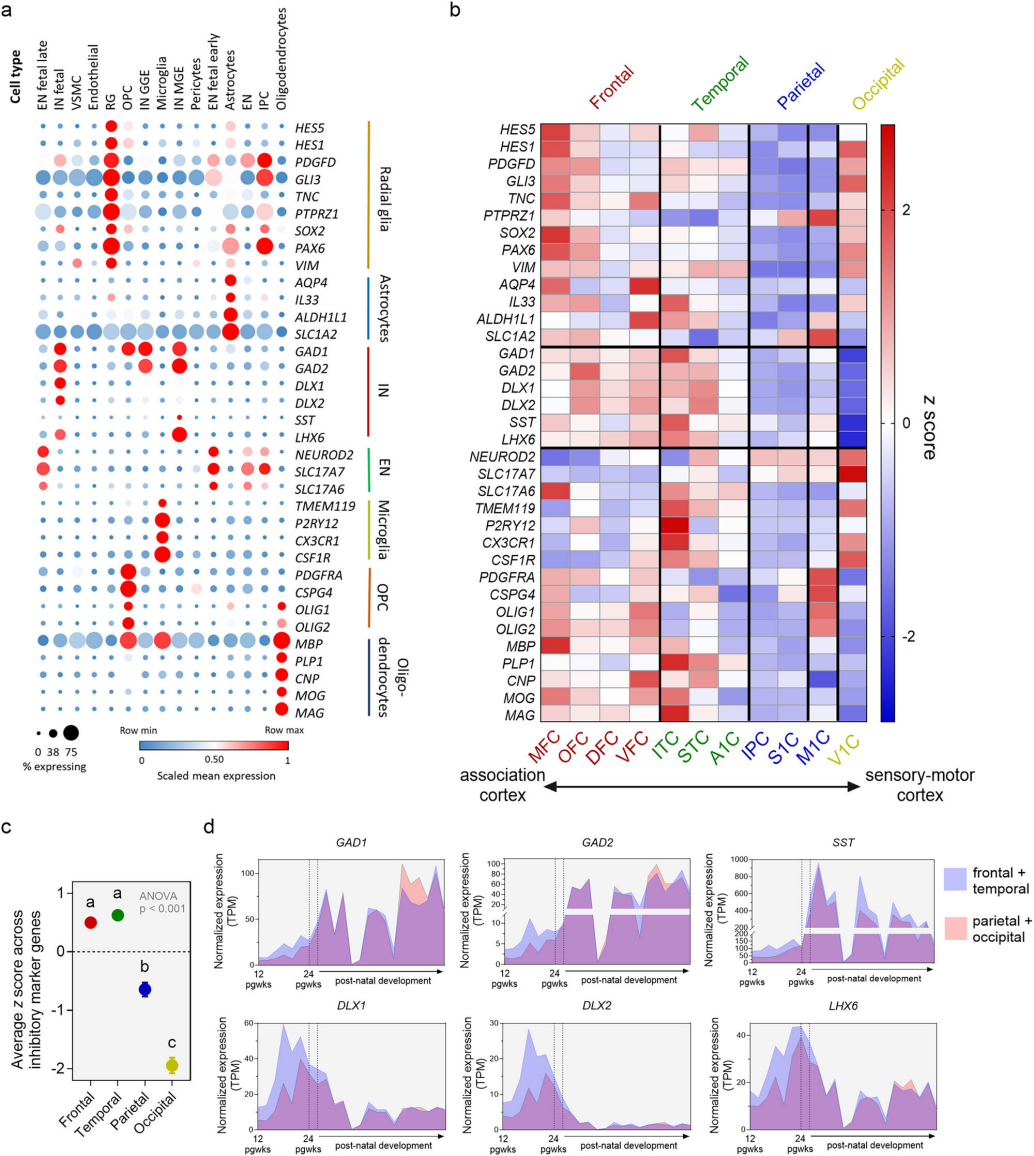
***a***, Marker genes show distinct expression for specific cell types. ***b***, ***c***, Higher expression of marker genes for inhibitory neurons (*GAD1*, *GAD2*, *DLX1*, *DLX2*, *SST*, *LHX6*) in frontal and temporal regions relative to parietal and occipital regions during early-to-mid fetal development (12–24 p.g. wks). ***d***, Normalized expression levels (TPM) of inhibitory neuron marker genes from 12 p.g. wks to adulthood (40 years) in the frontal + temporal (MFC, OFC, DFC, VFC, ITC, STC, A1C) versus parietal + occipital cortex (IPC, M1C, S1C, V1C). Inhibitory markers had higher expression levels in the frontal and temporal cortex during early-to-mid fetal development (12–24 p.g. wks), and this was limited to the fetal period, with no difference in expression in postnatal development (except *SST*).

Next, we investigated whether the observed increased connectivity between the fronto-temporal cortex and subcortical regions covaries with differences in abundance of specific cell types. We contrasted the expression levels of marker genes for different cell types between cortical regions (see Materials and Methods). As gene expression from only a limited number of cortical regions was available, and to facilitate the comparison with our connectivity analysis, we grouped all cortical regions into lobes before statistically comparing them. Marker genes are a set of genes with highly enriched expression in a particular cell type (Fig. 2*a*), and relative differences in their expression can be used to estimate differences in cell types across bulk RNAseq samples taken from different regions (Jew et al., 2020; Seidlitz et al., 2020; Mandal et al., 2021). Specifically, we explored expression differences in marker genes in the bulk RNA samples from the BrainSpan Developing Brain atlas for radial glia, astrocytes, inhibitory neurons, excitatory neurons, microglia, oligodendrocyte progenitor cells (OPCs), and oligodendrocytes.

We observed consistently higher expression across all inhibitory neuron markers (*GAD1*, *GAD2*, *DLX1*, *DLX2*, *SST*, *LHX6*), in regions within the frontal and temporal cortex relative to regions within the parietal, occipital, and motor cortex, ANOVA with FDR-corrected multiple comparisons: *F*_(3,20)_ = 146.8, *p* < 0.001 (Fig. 2*b*,*c*). As we had a limited number of regions across the lobes, it is difficult to ascertain with certainty whether the results reflect lobar differences or sensorimotor–association differences. However, the pattern of results may herald the early emergence of sensorimotor–association gradient: for example, anatomically the motor cortex is part of the frontal lobe, yet, the expression levels of inhibitory neuron markers align with that of sensorimotor regions. Similarly, across temporal regions (ITC, STC, A1C), the A1C (which is sensorimotor in adulthood in contrast to ITC) showed lower expression of inhibitory markers comparable to other sensorimotor regions (M1, S1, V1). The higher expression in the frontal regions of marker genes for inhibitory neurons was confined to the fetal period when inhibitory neurons have an excitatory function (Owens et al., 1996; Ben-Ari, 2002; Wang and Kriegstein, 2009; Murata and Colonnese, 2020), with little differences in postnatal expression (except *SST*; Fig. 2*d*). The pattern of higher expression in fronto-temporal regions holds valid both for markers whose expression was highest postnatally (*GAD1*, *GAD2*, and *SST*) as well as inhibitory markers with highest expression in the fetal period (*DLX1*, *DLX2*, and *LHX6*). There was also a statistically significant difference in expression levels across oligodendrocyte marker genes at *p* < 0.05, but the absolute levels of expression, as well as the differences, were very low (Supp Fig. 4).

### Increased plasticity in fronto-temporal cortical regions in adulthood

To be an integrative hub, a region does not only need to be well connected, but also to process the incoming information flexibly. Flexibility is a function of the plasticity of connections, as well as the collective functional properties of the cells comprising the region. To that end, we next investigated whether in adulthood high-level association regions maintain heightened state of plasticity to maximize integrative communication. Specifically, we explored differences across cortical regions in three processes directly related to plasticity: myelination and formation of PNNs—which repress plasticity—and markers of progenitor cells (stem cell-like states), which promote plasticity. We parcellated the brain with the Desikan–Killiany atlas and compared cortical myelination (T1w/T2w) and the expression levels of Myelin Basic Protein (*MBP*) as markers of mature myelin content across the adult cortex. PNNs are composed of the chondroitin sulfate proteoglycans neurocan, versican, brevican, and aggrecan which bond to hyaluronan (Fawcett et al., 2019). For assessing abundance of PNNs, we used the expression level of the gene *ACAN* encoding the proteoglycan aggrecan which is selectively expressed in PNNs (Rowlands et al., 2018). We focused on two broad categories of undifferentiated cell marker genes: (1) oligodendrocyte progenitor cells (OPCs) markers and (2) neural stem cells (NSCs) markers. The OPCs marker genes *ID4*, *SOX5*, *SOX6*, and *PDGFRA* have been shown to maintain OPCs in their undifferentiated state and repress myelin gene expression (Kondo and Raff, 2000; Wang et al., 2001; Stolt et al., 2006; Li et al., 2009; Zhu et al., 2014). The NSCs marker genes *SOX2*, *PAX6*, *HES1*, *HES5*, *VIM*, *NES*, and *GLI3* are expressed by immature progenitor cells of the nervous system (Zhang and Jiao, 2015; Vinci et al., 2016).

**Figure 3.**
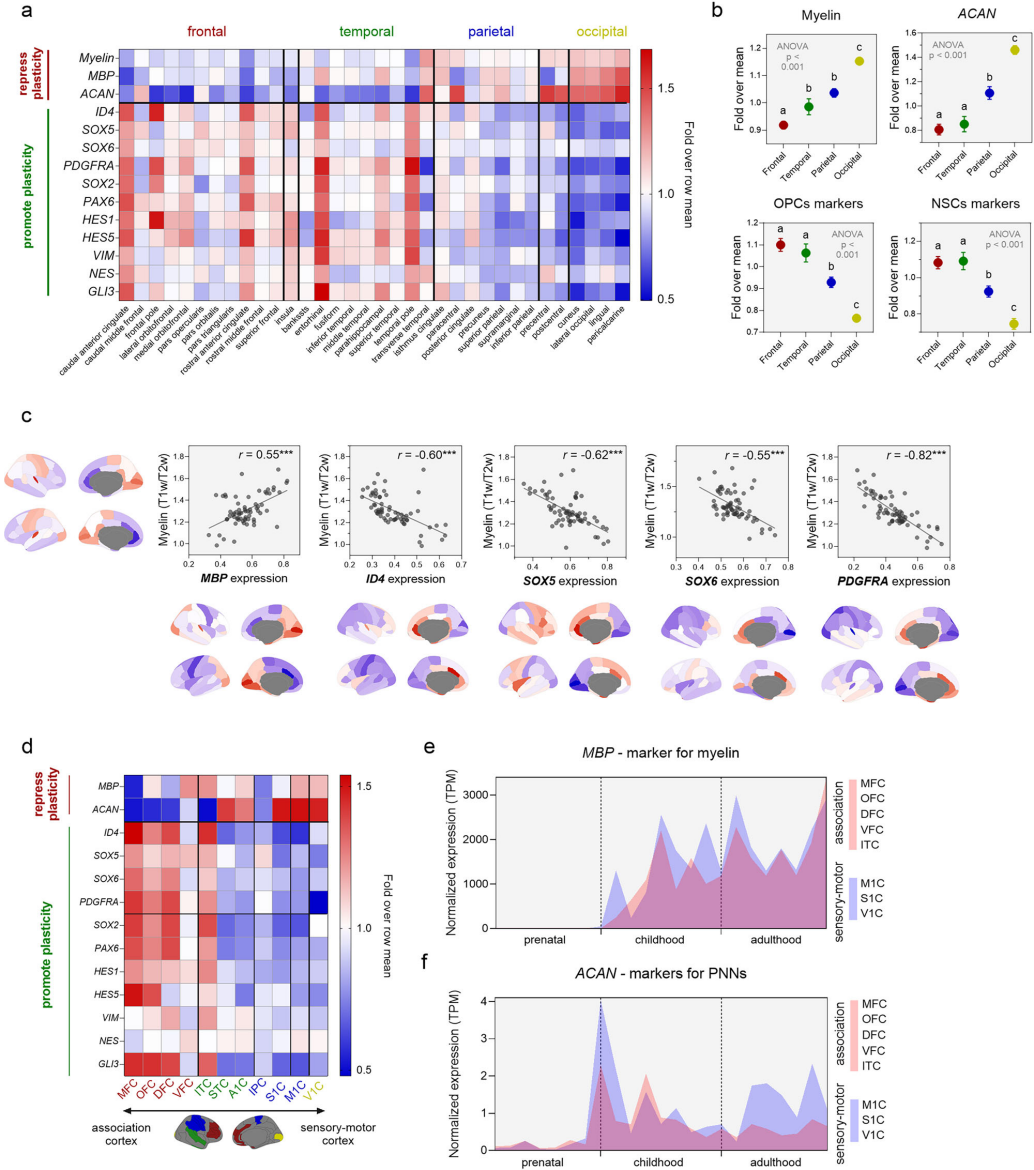
***a***, Cortical myelin content and relative gene expression levels across cortical regions (Desikan–Killiany atlas) of markers for myelin (*MBP*), perineuronal nets (*ACAN*), oligodendrocyte progenitor cells (OPCs; *ID4*, *SOX5*, *SOX6*, *PDGFRA*), neural stem cells (NSCs; *SOX2*, *PAX6*, *HES1*, *HES5*, *VIM*, *NES*, *GLI3*), and myelin abundance (top row). Values represent fold over row mean. ***b***, Regions across the frontal and temporal cortex have significantly lower myelin and expression of PNNs markers (which repress plasticity) but are enriched in processes promoting plasticity (OPCs and NSCs markers). ***c***, *MBP* expression positively correlates with cortical myelin content (T1w/T2w) across the cortex, whereas genes suppressing myelination (*ID4*, *SOX5*, *SOX6*, *PDGFRA*) correlate negatively. ***d***, Replication with an independent gene expression dataset (BrainSpan) in adult brains (18–40 years old). Association regions (frontal and inferior temporal cortex) are enriched in plasticity-promoting genes. ***e***, Normalized expression (TPM) of *MBP* as a marker for active myelination shows that association–sensorimotor difference emerges soon after birth and is most prominent during childhood. ***f***, In contrast, difference in PNNs (*ACAN* expression) was most pronounced in adulthood.

We show that cortical myelin content (T1w/T2w) and *MBP* expression were positively correlated (Fig. 3*c*) and both were lower in the frontal lobe relative to the rest of the cortex (Fig. 3*a*,*b*), while *ACAN* expression was significantly lower in the frontal and temporal compared with the parietal and occipital lobes (Fig. 3*a*,*b*). We further compared expression levels of genes promoting plasticity. Expression levels of the genes *ID4*, *SOX5*, *SOX6*, and *PDGFRA*, which are expressed by OPCs to maintain an undifferentiated state and suppress myelin production, were enriched in the frontal and temporal, relative to parietal and occipital regions (Fig. 3*a*,*b*). To assess whether the variation in cortical myelin (T1w/T2w) values across brain regions (DK atlas parcels) is related to the gene expression patterns for *MBP*, *ID4*, *SOX5*, *SOX6*, and *PDGFRA*, we performed a series of Pearson's correlations. There was a significant negative correlation between the level of expression of these myelin-suppressing genes and the cortical myelin content across the adult cortex (Fig. 3*c*). Finally, the expression of NSCs marker genes was also increased in frontal and temporal cortex (Fig. 3*a*,*b*). Overall, across all plasticity-related mechanisms analyzed here, with the exception of myelin content and *MBP* expression (which were highest in the frontal cortex), the frontal and temporal cortices were equally enriched in plasticity markers.

To investigate when during development do these differences in plasticity emerge, we used the developmentally enriched BrainSpan transcriptome atlas. We first replicated our findings of higher expression of plasticity-related processes in the frontal and temporal cortex in the samples of adult brains with the BrainSpan transcriptome atlas (Fig. 3*d*). Next, we grouped the regions into association (frontal cortex: MFC, OFC, DFC, VFC; inferior temporal cortex: ITC) and sensorimotor (M1C, S1C, V1C) and compared the expression across the lifespan for *MBP* and *ACAN* as marker for plasticity-repressing myelination and PNNs, respectively. Expression of *MBP* was higher in association relative to sensorimotor regions soon after birth (4 months old sample) and continued to differ during childhood through adolescence (until 19 years old), after which expression levels were similar (Fig. 3*e*). This is consistent with prior work showing that postnatally myelination proceeds along a sensorimotor–association gradient (Sydnor et al., 2023). In contrast, differences in expression of *ACAN* between association and sensorimotor regions emerged in late childhood/adolescence and were most pronounced in adulthood (Fig. 3*f*).

### Increased plasticity confers increased vulnerability to glioma

**Figure 4.**
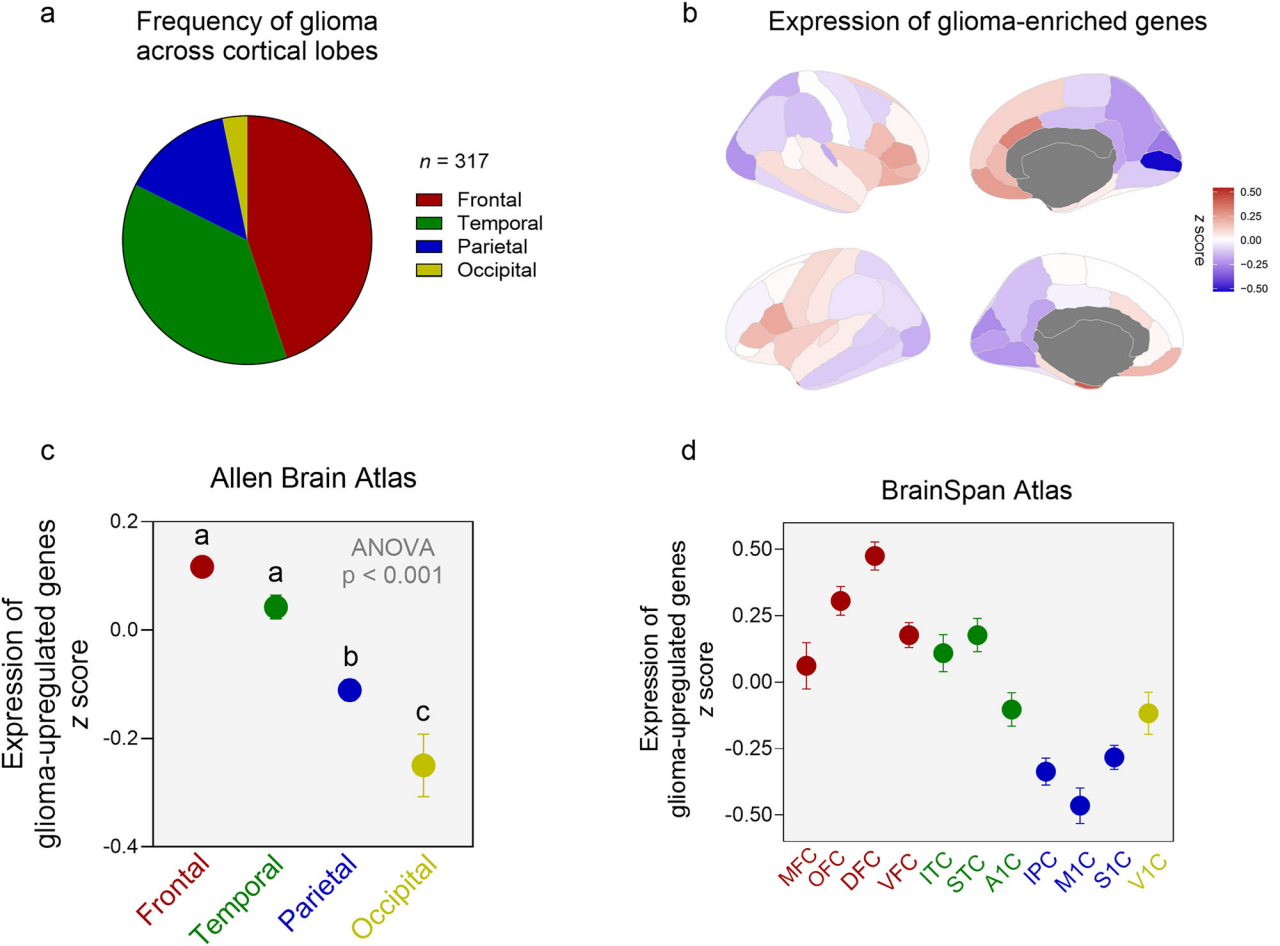
***a***, Frequency of glioma occurrences in adults across the four cortical lobes. ***b***, Relative expression levels of glioma-upregulated genes across cortical regions (Desikan–Killiany atlas). ***c***, The frontal and temporal cortex show significantly higher expression of glioma-upregulated genes in adult brains in the absence of glioma (Allen Human Brain Atlas). ***d***, Replication of results with six adults brain samples from the BrainSpan transcriptomic atlas.

We found that the frontal and temporal cortex are enriched in expression of NSCs and OPCs markers. This increased state of cellular plasticity led us to postulate that these regions will hold higher oncogenic potential and thus be more vulnerable to carcinogenesis as stems cells are the putative cells of origin in glioma (Alcantara Llaguno et al., 2009; Lee et al., 2018; Altmann et al., 2019). To that end, we compared the frequency of adult glioma across the cortex by collating previously published data (*n* = 317 cases; Larjavaara et al., 2007; Neftel et al., 2019; Scarpace et al., 2019). The distribution of gliomas across the cortex revealed an extraordinary imbalance: ∼45% of gliomas were found in the frontal cortex, ∼37% in temporal, ∼14% in parietal, and only ∼3% in the occipital lobe (Fig. 4*a*). To check whether the higher glioma frequency in frontal and temporal cortex remains after adjustment for the volume difference across lobes, we calculated a normalized glioma frequency by dividing the % frequency of glioma in each lobe by the number of voxels in that lobe. The frequency of gliomas per voxel in each lobe was as follows: frontal, 0.0016; temporal, 0.0023; parietal, 0.001, and occipital, 0.0004, indicating that he higher glioma frequency in frontal and temporal cortex remained after adjustment for their volume difference. The higher oncogenic potential of the fronto-temporal suggests that they might have a higher expression of genes typically expressed in gliomas. To test this, we used an existing database of genes which were upregulated in glioblastoma samples relative to normal brain tissue (Neftel et al., 2019; see Materials and Methods). Next, we mapped the expression of these glioma-upregulated genes across the cortex of adult brain samples using two brain transcriptomic atlases—the Allen Human Brain Atlas and (AHBA) and the adult samples from the BrainSpan Atlas. Across the two independent brain transcriptome atlases, the expression of glioma-upregulated genes was constantly higher in the frontal and temporal cortex relative to parietal and occipital cortical regions (Fig. 4*b*,*c*,*d*; AHBA: *F*_(3,1296)_ = 23.18, *p* < 0.0001; BrainSpan: *F*_(10,2310)_ = 20.96, *p* < 0.0001). This suggests that expression levels of glioma-upregulated genes across the cortex in the absence of glioma mirrors the cortical pattern of glioma frequency and underlying plasticity.

## Discussion

Here we leveraged fetal neuroimaging prior to myelination to investigate the connectivity signatures of cortical lobes. The frontal and temporal cortices were enriched in connections with the subcortex, potentially reflecting an early route to their establishment as integrative cortical centers. In adulthood, the fronto-temporal regions showed lower levels of myelination and expression of perineuronal nets markers and increased expression of progenitor cells markers, suggesting a heightened state of plasticity. Together, our results suggest that the early establishment of subcortical connections and prolonged maturation might enable the association fronto-temporal cortex to flexibly integrate information and support increasingly abstract representations.

What are the mechanisms leading to the higher subcortical connectivity of the fronto-temporal cortex? A possible mechanism might be through GABA signaling. Using gene expression from fetal brains, we report higher expression of inhibitory neuron marker genes in regions of frontal and temporal cortex compared with parietal and occipital regions. This finding has been demonstrated previously (Al-Jaberi et al., 2015; Molnár et al., 2019) and is thought to reflect a genetically determined preferential inhibitory neuron generation in the fronto-temporal cortex (Al-Jaberi et al., 2015). GABA is the main inhibitory neurotransmitter in the brain and acts primarily by binding to GABA_A_ or GABA_B_ receptors which is critical for the development of cortical circuits (Peerboom and Wierenga, 2021). While GABA neurons are inhibitory in the postnatal brain, they have an excitatory function during fetal cortical development (Owens et al., 1996; Ben-Ari, 2002; Wang and Kriegstein, 2009; Murata and Colonnese, 2020), with a shift toward their established inhibitory function at around the first postnatal week in humans (Kilb, 2012). Crucially, GABA inhibitory neurons have been demonstrated to preferentially generate action potentials in pyramidal neurons of layers V and VI of the immature cortex (Rheims et al., 2008), which are the layers that form corticofugal projections to subcortical regions such as the basal ganglia and thalamus (Baker et al., 2018; Usrey and Sherman, 2019), providing a potential mechanism for the covariance between subcortical connections and the number of inhibitory neurons across the developing cortex.

We restricted the connectivity analysis only to the narrow 22–25 p.g. weeks age range in order to avoid the effect of myelination on connectivity estimates in older subjects and thus, derive a measure of connectivity based solely on axonal tracts. Connectivity strength of a given region after the start of myelination likely reflects a combination between the number of axonal connections and their myelination levels; however, their relative contribution to the strength of connectivity measured is unclear (Oldham and Fornito, 2019). As such, determining which cortical regions are connectivity “hubs” throughout the lifespan would be influenced by the relative prevalence of axonal connections and myelination in these regions, which itself varies temporally and spatially. For example, the occipital lobe does not have as many early connections as the frontal lobe; however, it is one of the first cortical regions to myelinate which would overestimate its connectivity in the early postnatal period. In contrast, association regions tend to be the last ones to myelinate which would overestimate their relative gain of connections during the adolescence–adulthood transition. Indeed, a shift in “hubness” of regions between primary and association regions has been documented during the transition from neonatal to childhood/adolescence periods (Oldham and Fornito, 2019). However, we recognize that the structural connectivity we measured during 22–25 p.g. weeks window may not necessarily be representative of future connectivity as there are both further extension of axons as well as retraction of exuberant axons during the late gestational and early postnatal periods (LaMantia and Rakic, 1990; Innocenti and Price, 2005).

The finding that the frontal lobe has more connections in the 24-week fetal brain is somewhat counterintuitive to the fact that its connections are the last to myelinate (see also Lebel et al., 2008; Baum et al., 2022; Sydnor et al., 2023). We propose that it is precisely the combination of early overconnectivity and later myelination that enables the frontal cortex to serve its function as an integrative hub supporting increasingly abstract levels of representation throughout the human lifespan. There are two main properties that a cortical region needs to satisfy in order to perform flexible integration of information: (1) connect to multiple regions and (2) keep long-term plasticity of these connections to incorporate changing inputs at differing rates from other regions as they mature. To this end, our results demonstrate that the frontal cortex is particularly enriched in connections to the subcortical regions, which are hubs in the brain (Oldham and Fornito, 2019). This is consistent with the previously reported increased functional connectivity of the frontal cortex during fetal development (Karolis et al., 2023). In line with the second property, the frontal cortex develops myelination of its connections last and has less myelin compared with other cortical regions even when fully matured in adulthood. Although myelination contributes to faster information transfer and therefore serves a more efficient communication, it is also one of the processes that inhibits brain plasticity and closes critical periods (McGee et al., 2005; Hübener and Bonhoeffer, 2014). Thus, by setting up early connections and allowing for these connections to be plastic (less myelinated) over a longer time, the frontal cortex may flexibly integrate information. The question of what factors delay the formation of myelin to the frontal region postnatally, and more broadly, what factors underlie the extraordinary postnatal neoteny of the frontal cortex, constitutes a most promising field for future investigations.

Over two independent transcriptome datasets, we found that in adulthood the association fronto-temporal cortex remains more plastic as it harbored the lowest levels of marker genes for PNNs. Although much less is known about differences between cortical regions in the abundance of PNNs in the human brain, prior work in the adult rat cortex suggests that association regions have less PNNs compared with sensorimotor region (Galtrey and Fawcett, 2007). Our transcriptomic results suggest that this spatial pattern might be conserved in the adult human brain and that differences between sensorimotor and association regions become most prominent in adolescence/adulthood, although histological verification would be required. Consistent with the idea of increased plasticity, the fronto-temporal cortex also expressed higher levels of marker genes for undifferentiated progenitor cells (OPCs and NSCs). Here we want to emphasize that our results are agnostic to the exact process contributing to the increased gene markers for progenitor cells. Unlike neurons, OPCs continue to differentiate throughout adulthood (Crawford et al., 2014); thus, the OPCs markers likely reflect yet undifferentiated oligodendrocyte progenitors. However, it is well established that there is no neurogenesis in the adult cortex (Rakic, 1985; Kornack and Rakic, 2001; Spalding et al., 2005), suggesting that the NSCs markers expression does not stem from bona fide neuron progenitors. As cells can exist in different “states” (Trapnell, 2015), one possibility is that the NSCs markers are expressed by mature, differentiated neurons to drive a more plastic cellular state. Another alternative is that the increased expression of NSCs markers captures a state of dedifferentiation where mature cells gradually lose their differentiation and transform into stem cells (Yang et al., 2010; Corti et al., 2012; Mills et al., 2019). The blurred borders between cell types and cell states have been discussed extensively elsewhere (Trapnell, 2015; Mills et al., 2019). Here, we seek to highlight that whether the increased cellular plasticity is attributed to truly undifferentiated progenitor cells, or mature cells acquiring more plastic stem-like cellular states, our results, nevertheless, suggest that the association cortex harbors more plastic cellular potential.

When observing regional differences in plasticity across the cortex, one specious conclusion is to view the regions with higher plasticity in some sense as exceptional or “high-level.” This is particularly tempting in light of the well-established association between the frontal cortex and complex cognitive abilities, as well as its disproportional evolutionary expansion in humans (Miller et al., 2002). We want to stress that the appropriate development and functionality of the association cortex, as well as the execution of complex cognitive tasks, necessarily rely on stable inputs from less plastic regions. In this view, plastic responses could be maladaptive when stable signals are required. In other words, to support intelligent (adaptive) behavior, both stable and plastic neural responses are of equal value. Differences between individuals in the developmental timing and extent of processes driving plasticity (myelination, PNNs formation) are propitious candidate mechanisms for investigating individual differences in cognition and behavior.

Finally, we linked typical physiology to pathology by showing that greater plasticity confers greater oncogenic vulnerability. Our analysis, as well as extensive previous work (Larjavaara et al., 2007; Altmann et al., 2019; Mandal et al., 2021; Romero-Garcia et al., 2023), demonstrated that glioma involving the cortex is preferentially located in the fronto-temporal regions relative to other cortical regions. This pattern is specific to gliomas as metastases to the cortex from non-neural primary cancers do not show the same cortical distribution (Kwon et al., 2020; Cardinal et al., 2022; Bonert et al., 2023). Stem cells are the likely cells of origin in glioma (Alcantara Llaguno et al., 2009; Lee et al., 2018; Altmann et al., 2019), and differences in their abundance across the cortex have been linked to the different rates of gliomas across the cortex (Mandal et al., 2021; Romero-Garcia et al., 2023). As gliomagenesis is a probabilistic event, the observation that the frequency is higher in fronto-temporal regions suggests that stem cells might be disproportionately supplied in those regions in adulthood, relative to regions in the parietal and occipital lobes, which is in line with our findings of increased stem cell-like expression. However, if gliomagenesis was only a function of the abundance of stem cells, one would expect higher glioma rates in utero and early postnatal life. Given that the peak incidence of glioma is between 45 and 75 years of age (Altmann et al., 2019), the higher glioma frequency in the association cortex would likely involve immune and inflammation factors [which may themselves be higher in frontal cortex due to the increased metabolic rate there (Castrillon et al., 2023)], independent of stem cells abundance. Our results contribute to the literature by showing that fronto-temporal cortical regions express higher levels of genes upregulated in glioma even in the absence of malignancy, suggesting that their higher oncogenic potential is, at least in part, due to greater glioma-like normative expression state, potentially providing more conductive environment for gliomagenesis, progression, and survival.

In conclusion, we demonstrated that at 24 postgestational weeks fronto-temporal regions are disproportionally connected to subcortical regions, highlighting their role in early integrative cortical-subcortical communication. In adulthood, the fronto-temporal cortex had lower myelin content, lower markers of perineuronal nets, and increased markers of undifferentiated progenitor cells, suggesting heightened plasticity of its connections and cellular state. However, the association regions showed an increased incidence of gliomas, as well as expression of glioma-associated genes in the absence of disease, suggesting that the heightened plasticity confers greater oncogenic vulnerability. Together, our results provide evidence of divergent patterns of connectivity in utero, and plasticity in adulthood between cortical lobes and support a framework which views functional differences across cortical regions as manifestations of differences in connectivity and their plasticity.

## Data Availability

Codes and results from the current analyses are publicly available at https://osf.io/s4qb2/.
